# Find the real responders and improve the outcome of awake prone positioning

**DOI:** 10.1186/s13054-021-03663-w

**Published:** 2021-07-08

**Authors:** Heyan Wang, Hangyong He

**Affiliations:** 1 Department of Critical Care Medicine, The Sixth Hospital of Guiyang, Guiyang City, Guizhou Province China; 2 Department of Respiratory and Critical Care Medicine, Beijing Institute of Respiratory Medicine, Beijing Chao-Yang Hospital, Capital Medical University, No. 8 Gongren Tiyuchang Nanlu, Chaoyang District, Beijing, 100020 China

## Dear editor,

We read with great interest of the report by Rosen and colleagues [1] about the effect of awake prone positioning (APP) on intubation rate among patients with moderate to severe hypoxemic respiratory failure (HRF) due to COVID-19. They identified a implemented protocol for APP increased duration of prone positioning, but did not reduce the rate of intubation compared to standard care. From our point of view, some details about whether APP could decrease intubation in patients with HRF due to COVID-19 need to be further clarified.

First, in Rosen’s study [1], the patient in standard care group also received a median prone duration of 3.4 h compared with 9.0 h per day in the prone group, which means that the real intervention factor they investigated was whether a prolonged APP could further decrease intubation rate. Therefore, it may be more appropriate to draw a conclusion that prolonged APP did not reduce the rate of intubation in patients with HRF due to COVID-19.

Second, Rosen and colleagues [1] reported a 33% intubation rate in both groups, which is much lower than that reported in two other studies [2, 3] with similar baseline characteristics, degree of respiratory failure but shorter APP duration (41% and 49% in the control group and 40% and 58% in the prone group) compared with their investigation. Based on this data, could we speculate that prolonged APP does decrease the intubation rate?

Third, in Rosen’s study [1], most patients were put on APP when they were supported with noninvasive ventilation, which is a higher level support than high flow nasal canula (HFNC) [4]. This may indicate that they use the APP more like a “rescue” therapy rather than an “adjunctive” therapy in a late stage of ARF, which may get less response to APP and therefore less difference in intubation rate.

In summary, combining this lack of accuracy of comparison of APP and non-APP patients, and the possibly late initiation of APP in the moderate to severe ARF COVID-19 patients, a priori chance of finding a difference in intubation was low and may explain the negative findings for the APP. And further analysis with data from comparison of non-APP populations and prolonged APP group, especially in patients support with HFNC and prolonged APP, is needed for a more settled conclusion.

## Data Availability

Not applicable.
